# Development of a Molecularly Imprinted Polymer-Based Sensor for the Electrochemical Determination of Triacetone Triperoxide (TATP)

**DOI:** 10.3390/s141223269

**Published:** 2014-12-05

**Authors:** Samuel Kassahun Mamo, Jose Gonzalez-Rodriguez

**Affiliations:** School of Chemistry, University of Lincoln, Brayford Pool, Lincoln LN6 7TS, UK

**Keywords:** TATP, explosives, forensic science, electrochemistry, molecular imprinted polymer, IED, trace analysis

## Abstract

The explosive triacetone triperoxide (TATP), which can be prepared from commercially readily available reagents following an easy synthetic procedure, is one of the most common components of improvised explosive devices (IEDs). Molecularly-imprinted polymer (MIP) electrochemical sensors have proved useful for the determination of different compounds in different matrices with the required sensitivity and selectivity. In this work, a highly sensitive and selective molecularly imprinted polymer with electrochemical capabilities for the determination of TATP has been developed. The molecular imprinting has been performed via electropolymerisation onto a glassy carbon electrode surface by cyclic voltammetry from a solution of pyrrole functional monomer, TATP template and LiClO_4_. Differential Pulse Voltammetry of TATP, with LiClO_4_ as supporting electrolyte, was performed in a potential range of −2.0 V to +1.0 V (*vs.* Ag/AgCl). Three-factor two-level factorial design was used to optimise the monomer concentration at 0.1 mol·L^−1^, template concentration at 100 mmol·L^−1^ and the number of cyclic voltammetry scan cycles to 10. The molecularly imprinted polymer-modified glassy carbon electrode demonstrated good performance at low concentrations for a linear range of 82–44,300 μg·L^−1^ and a correlation coefficient of r^2^ = 0.996. The limits of detection (LoD) and quantification (LoQ) achieved were 26.9 μg·L^−1^ and 81.6 μg·L^−1^, respectively. The sensor demonstrated very good repeatability with precision values (*n* = 6, expressed as %RSD) of 1.098% and 0.55% for 1108 and 2216 μg·L^−1^, respectively. It also proved selective for TATP in the presence of other explosive substances such as PETN, RDX, HMX, and TNT.

## 1. Introduction

TATP (triacetone triperoxide, or 3,3,6,6,9,9-hexamethyl-1,2,4,5,7,8-hexaoxanonane) is a peroxide explosive that has become increasingly popular among terrorists due to the ready availability of its starting materials and a rather simple, although highly dangerous, synthesis procedure. TATP is a primary explosive, but has a secondary explosive velocity of detonation of 5.3 km·s^−1^ which is 80% as powerful as TNT. It is a white, water insoluble powder which can be synthesized from chilled acetone and hydrogen peroxide in the presence of concentrated acids such as sulphuric and hydrochloric acid [1]. In the past decade several terrorist attacks or attempts have been directly linked with the use of TATP as the main explosive or as part of Improvised Explosive Devices (IEDs) [2,3].

Detection of TATP is a challenge as the conventional explosive detection devices and canine detection methods, commonly used in public terminal controls, depend on the presence of nitro groups and metallic elements, which this explosive does not have in its structure [3]. TATP has a quite unsuspicious appearance, with no significant UV-Vis absorption or fluorescence properties [1]. Furthermore, as IEDs based on TATP have been used on many occasions by suicide bombers, portable detection methodologies are in great demand.

Infrared (IR) and Raman spectroscopic methods have been used as portable, fast and reliable methods for the detection of TATP and hexamethylenetriperoxide diamine (HMTD) [4]. However, both IR and Raman spectroscopic methods lack sensitivity and are usually unable to discriminate peroxide-based explosives from innocuous peroxides used in various laundry detergents [5].

Ion mobility spectroscopy (IMS) has also been used to detect TATP dissolved in toluene. Buttigieg *et al.* [6] achieved good results for a rapid, on-site detection method with relatively good detection limits (187 μg·mL^−1^) for trace analysis. However, the method presented safety limitations associated with the possibility of detonations inside the flight tube.

Both liquid and gas chromatography coupled to mass spectrometry have been used for trace analysis of TATP and offered excellent detection limits. Kende *et al.* [1] achieved a LoD of 5 ng·mL^−1^ using a GC-MS system, whereas Widmer *et al.* [7] reported a LoD of 10^−7^ mol·L^−1^ using a LC-MS method. The main shortcomings of these methods are the need for sample pretreatment, low portability and, in the case of GC-MS, the possibility of sample loss due to thermal decomposition of TATP in the injector port.

Chemical sensors offer a good solution to fulfill the portability requirement in those cases where selectivity and sensitivity are good enough. For example, immunosensors using anti-TATP antibodies [8] presented relatively high detection limits (840 μg·L^−1^) and good selectivity, although this was hindered by the absence of characteristic side chains and/or nucleophilic substituents usually required for excellent immunological recognition. The varying conformations of the TATP 3-D structure, from the more stable twisted boat-chair to the least stable twisted chair-chair conformation, also affected the specificity of the linkage. Immunosensors also lack robustness and stability in extreme field conditions. Piezoelectric sensors based on quartz crystal microbalances have also been employed. They have detection limits lower than 6.6 μg·L^−1^ due to the low capacity of the thin polymer films and they offer the advantage of vapour analysis of TATP [9,10].

Electrochemical sensors can provide an alternative for field determination of TATP as they are portable, highly selective, highly sensitive, fast, and inexpensive. However, most of the electrochemical systems found in the literature are based on indirect determinations that compromise the selectivity and sensitivity of these sensors [3].

Amperometric sensors based on glassy carbon electrodes modified with Prussian Blue have been used to detect H_2_O_2_ produced after the photochemical degradation of TATP using a laser. Very low LoDs were achieved with this method (50 nmol·L^−1^) but it proved prone to interferences from other sources [11]. Chronoamperometric detection using the electrocatalytic reduction of TATP with the Fe(II/III)EDTA complex on a glassy carbon electrode has also been reported. The method is highly pH-dependent and it is also based on the indirect analysis of the H_2_O_2_ produced [12].

A very selective detection method using the unique reaction of TATP with bromides to yield bromoacetones was used for a two-step chronoamperometric detection method using glassy carbon electrodes [13]. This method demonstrated good linearity for the concentration range of 0 to 100 μmol·L^−1^, with a limit of detection similar to other electrochemical methods employed for TATP detection.

The use of molecular imprinted polymers (MIPs) in conjunction with electrochemical sensors, such as the one proposed in this work, can offer highly selective and sensitive miniaturised sensors based on template recognition for forensic applications such as the analysis of drugs and explosives. This approach has already been developed in the analysis of other explosives, such as TNT [14]. To the best of our knowledge, there are no reported papers in the literature on the use of electrochemical sensors modified with MIPs for the direct determination of TATP. As demonstrated by the results of this work, the selective determination of TATP was achieved even in the presence of other explosives likely to be found in explosive mixtures previously used by terrorists.

## 2. Materials and Methods

### 2.1. Chemicals

Pyrrole (98% reagent grade) from Sigma-Aldrich (Suffolk, UK), lithium perchlorate (≥98% purity) from Fluka (Suffolk, UK) and acetonitrile (HPLC grade) from Fisher Scientific (Loughborough, UK) were used for the electropolymerisation procedure. Phosphate buffer tablets (pH = 7), potassium chloride and potassium monohydrogen phosphate, used in template removal were purchased from Fisher Scientific. Sulphuric acid, potassium hydroxide and potassium chloride used in the cyclic voltammetry cleaning of the bare glassy carbon electrode were all purchased from Fisher Scientific. Fumed silica (particle size 0.007 μm) and aluminium oxide (activated and neutral) used for polishing the glassy carbon electrodes were both purchased from Sigma-Aldrich. Nitric acid (analytical reagent grade), sulphuric acid (analytical reagent grade), methanol (HPLC grade) and ethanol (analytical reagent grade) all purchased from Fisher Scientific, were used to clean the polished glassy carbon electrode. For the preparation of triacetone triperoxide explosive powder, hydrogen peroxide (>30% w/v, laboratory reagent grade), acetone (analytical reagent grade), hydrochloric acid (37% w/v, analytical reagent grade), acetonitrile and HPLC grade methanol were used and all purchased from Fisher Scientific. Pentaerythritol tetranitrate (PETN), 2,4,6-trinitrotoluene (TNT), 1,3,5-trinitroperhydro-1,3,5-triazine (RDX), and octahydro-1,3,5,7-tetranitro-1,3,5,7-tetrazocine (HMX) standards (purity > 99.99%) used in selectivity studies were all purchased from Ultra Scientific (Middlesex, UK).

### 2.2. Instruments and Apparatus

All electrochemical experiments and measurements were performed using a Metrohm 757 VA Computrace (Metrohm UK Ltd, Chesire, UK) connected to a personal computer (Compaq DeskPro, Windows 95, (Compaq, Harris, TX, USA) using Ct757 v.1 software from Metrohm (Chesire, UK). A three electrode system was used, using Ag/AgCl as a reference electrode, platinum as a counter electrode and a glassy carbon electrode with an area of 0.126 cm^2^ as a working electrode, all purchased from Metrohm. The purity of the laboratory synthesised triacetone triperoxide explosive powder samples was checked using a Clarus 600 fast GC-MS with Turbomass 6.0 software (PerkinElmer, Waltham, MA, USA). A PerkinElmer Spectrum 100 FT-IR spectrometer was also used to obtain the IR spectra of the synthesised TATP. The TATP explosive powder and other solid reagents were weighed using an AND analytical balance (A&D instruments, Abingdon, UK).

### 2.3. Synthesis of TATP

The synthetic procedure was adapted to manufacture less than one gram of TATP so that the danger of any possible explosion was reduced. A 30% solution of chilled H_2_O_2_ (0.68 mL) and chilled acetone (0.95 mL) were kept in a salted ice bath at −5 °C in a 100 mL beaker. Chilled concentrated HCl (a total of 240 μL) was added in 20 μL aliquots every 4 min. Concentrated HCl was added carefully and slowly to avoid a vigorous reaction, which may lead to an explosion. All work was performed in a fume cupboard and the necessary protective equipment worn at all times and according to UK legislation for handling explosive materials. All potential sources of ignition were eliminated in the working area and stirring of the solutions was performed carefully to avoid any friction that could have provoked an explosion. Complete precipitation of TATP crystals was achieved by refrigerating overnight at 4 °C in a refrigeration unit suitable to store explosive substances. The white crystals of solid TATP were redissolved in methanol and recrystallised by addition of deionised water. The solid TATP powder was then filtered, washed again with deionised water and dried with nitrogen. The powder was immediately dissolved in acetonitrile, an anoxic solvent, to minimise the risk of explosion.

The purity of the synthesised TATP was calculated to be 98.6% and checked by comparing the mass spectrum with a 2008 NIST MS library and the FT-IR spectrum with the IR-NIST library. The concentration of the solutions was also checked using these techniques and stock solutions prepared.

### 2.4. Preparation of the Molecular Imprinted Polymer on the Electrode

A three factor two level randomised factorial design with one-centered-point was performed using STATGRAPHICS Centurion XVI statistical software (StatPoint Technologies Inc., Warrenton, VA, USA) to optimise the template concentration, the monomer concentration and the number of scan cycles to be used in the electropolymerisation using a surface response representation.

Prior to the molecular imprinting by electropolymerisation, the glassy carbon electrode was polished to a mirror-like surface successively with activated aluminium oxide and 0.007 μm silica slurry. The polished electrodes were sonicated for 5 min in each of concentrated nitric acid, concentrated sulphuric acid, ethanol, methanol and acetonitrile solutions sequentially and dried using nitrogen gas. The aim of this treatment was to clean the surface and eliminate any contaminants that could inhibit the polymerisation process. The polished and sonicated bare glassy carbon electrode was first subjected to five cycles of cyclic voltammetry at a scan rate of 0.1 V·s^−1^ and a potential range from −0.1 to +1.5 V in a 0.05 mol·L^−1^ sulphuric acid solution. After this, five cycles with a potential range from −1.2 to +0.2 V in a 1.0 mol·L^−1^ KOH solution and another five extra cycles from −1.0 to +1.0 V in a 0.1 mol·L^−1^ KCl solution were carried out.

The electropolymerisation was achieved in an electrolyte solution which contained 0.1 mol·L^−1^ pyrrole functional monomer, 0.1 mol·L^−1^ LiClO_4_ supporting electrolyte and 100 mmol·L^−1^ TATP template in acetonitrile as a porogenic solvent. The polymerisation of pyrrole and entrapment of TATP into the polymer lattice was performed by cyclic voltammetry using a potential range of −1.0 V to +1.0 V at a scan rate of 0.05 Vs^−1^ for 10 scan cycles, after initial purging of the mixture with nitrogen gas for 300 s. After this, a single conditioning cycle from −1.0 V to +1.0 V followed by 5 min equilibration of the working electrode was carried out.

Template removal was achieved using two electrochemical steps. The first step consisted of three cycles using a potential range of −1.0 V to +1.4 V at a scan rate of 0.05 V·s^−1^ in an electrolyte solution containing 0.1 mol·L^−1^ KCl and 0.05 mol·L^−1^ phosphate buffer solution. In the second step a fixed potential of +1.4 V as applied to the electrode in a 0.2 mol·L^−1^ K_2_HPO_4_ solution for 10 min. The molecular imprinted glassy carbon electrode was then dried with nitrogen gas for 5 min and was then ready to use.

### 2.5. Voltammetric Analysis

Differential pulse voltammetry (DPV) analyses were performed in order to obtain quantitative results for a set of acetonitrile-based solutions containing TATP at different concentrations and using 0.025 mol·L^−1^ LiClO_4_ as the supporting electrolyte. Nitrogen gas was bubbled through the sample for 5 min and a single conditioning cycle using a potential range of −1.0 V to +1.0 V was run, followed by 5 min equilibration. The TATP solutions were analysed using a scan rate of 15 mV·s^−1^, a modulation amplitude of 50 mV, and a step potential of 8 mV in a scanning potential range of −2.0 to +1.0 V.

## 3. Results and Discussion

**Figure 1.**
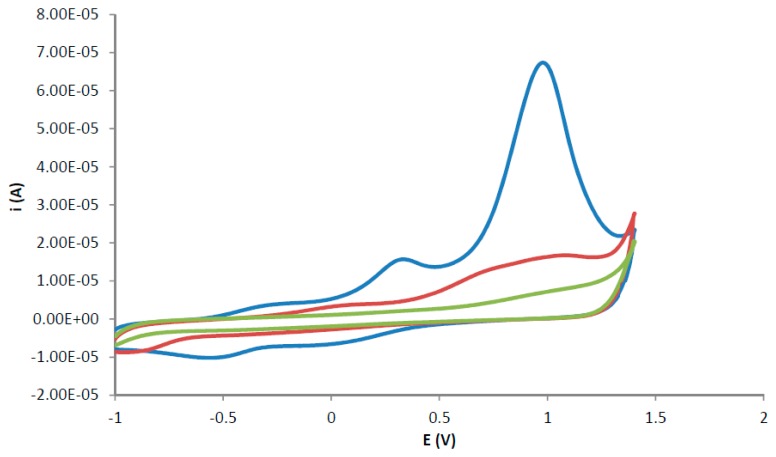
Cyclic voltammograms of the template removal using an aqueous solution of 0.05 mol·L^−1^ KCl and phosphate buffer (pH = 7.0) at a scan rate of 0.05 V·s^−1^ and 3 scan cycles (blue-3 scans; red-2 scans; green 3 scans).

The removal of the template from the polymer matrix was carried out using three cyclic voltammetry cycles, as described in the first step of the template removal procedure in the materials and methods section. The removal of the template from the polymer matrix was observed as decreasing anodic current peaks at +1.2 V for successive cyclic voltammetric cycles, as shown in Figure 1. TATP trapped in the polymer lattice oxidises under the aqueous conditions described. The molecule breaks down leaving the recognition site ready for another TATP molecule to be inserted (a process that will happen in the analysis step).

The fixed over-potential in the second step of the initial removal process enabled the creation of more stable and rugged imprinted sites by strengthening the polymer network [15].

### 3.1. Optimisation of the Variables

A three-factor two-level factorial design was used for the optimization of the template concentration, functional monomer concentration and the number of scan cycles as independent variables using the intensity of the electrochemical process as response variable. The high and low values chosen were: for the template concentrations, 100 and 20 mmol·L^−1^; for the functional monomer concentrations, 0.1 and 0.05 mol·L^−1^ and for the number of scan cycles 10 and 40, respectively. This design studied the effect of the three factors in 18 runs in 2 blocks, with each block including one centre point. The centre points of the design was run with a template concentration of 60 mmol·L^−1^, a functional monomer concentration of 0.075 mol·L^−1^, and 25 scan cycles. The order of the experiments has been fully randomized as this will provide protection against the effects of lurking variables.

The optimisation of the main variables affecting the electro-polymerisation process to achieve maximum sensitivity without compromising the selectivity of the polymer is critical. The main variables affecting the building process are the number of cycles, monomer concentration and template concentration [16]. The number of scan cycles in the electro-polymerisation process further affects the sensitivity and linearity of the molecular imprinted polymer sensor produced. A lower number of voltammetric cycles exhibited favourable analytical performance whereas a higher number of voltammetric cycles led to the formation of a thicker sensing film with less accessible imprinted sites, as observed by Sharma *et al.* [16].

The rate of polymer nucleation and film growth can be controlled by optimising the concentration of the monomer used. A very low functional monomer concentration led to a lower rate of polymer nucleation and film growth producing a non-uniform polymer film. Whilst conversely, a very high monomer concentration led to very thick polymer films as the rate of polymer growth was higher per each scan cycle [16].

**Figure 2.**
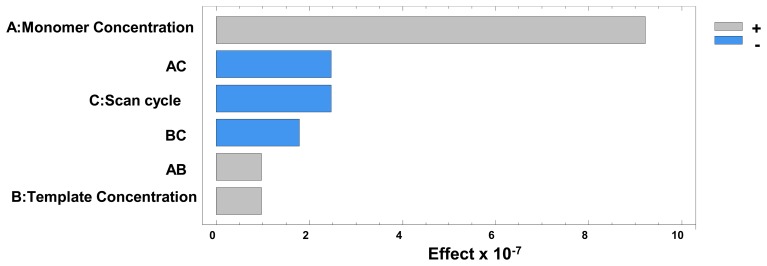
Pareto chart obtained in the optimisation of the electropolymerisation process.

**Figure 3.**
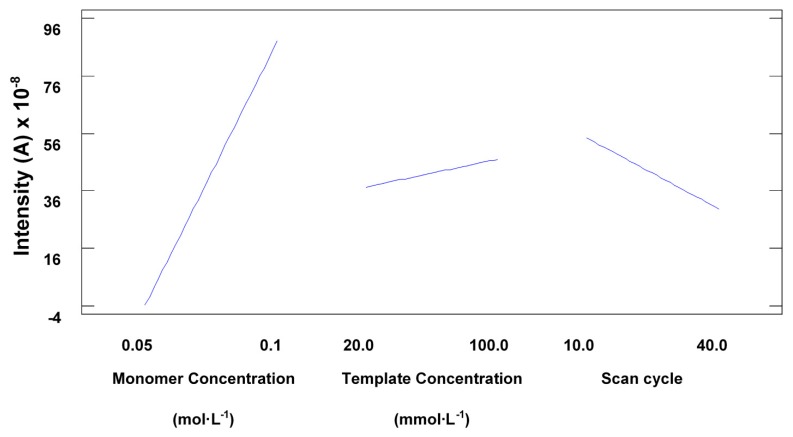
Main effects plot showing the effect of low and high values for the main variables on the intensity of the current.

According to Sharma *et al.*, sensitivity increases with higher template concentrations as this enables the creation of a higher number of imprinted sites on the polymer surface. However, this also allows the formation of irregularly shaped cavities which leads to a decrease in selectivity. In our study, the Pareto chart (Figure 2) and the main effects plot obtained in the optimisation of these variables (Figure 3) showed that the pyrrole concentration was the main factor affecting the sensitivity of the sensor.

**Figure 4.**
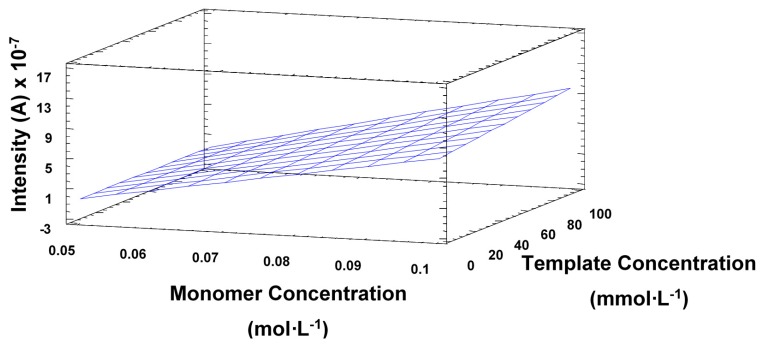
Representation of the surface response of the optimization process for the production of a TATP-MIP sensor for the template and monomer concentration.

The experimental design also showed that the template concentration did not have a very significant effect on the electrode response, which seemed to imply that the selectivity would not be compromised, as further demonstrated during the method validation. Statistical analysis also proved that an increase in the number of cycles also implied a significant decrease in sensitivity, as seen in Figure 3. This is consistent with the observations made by Sharma *et al.*, the surface response (Figure 4) also shows the main effect in the intensity comes from the increasing concentration of monomer.

An experimental equation from the ANOVA analysis of the three main independent variables (number of cycles and both pyrrole and TATP concentrations) *versus* the intensity (A) was produced and the optimum values obtained for the different variables of interest can be found in the Experimental section:

$$I\left(A\right)=-1.4523\times 10^{-6}+2.38042\times 10^{-5}\left[\text{Pyrrole}\right]+1.29479\times 10^{-9}\left[\text{TATP}\right]+2.54458\times 10^{-8}SC+4.8375\times 10^{-8}\left[\text{Pyrrole}\right]\left[\text{TATP}\right]-3.30667\times 10^{-7}\left[\text{Pyrrole}\right]SC-1.48542\times 10^{-10}\left[\text{TATP}\right]SC$$

where [pyrrole] = mol·L^−1^, [TATP] = mol·L^−1^, SC = number of scan cycles.

The optimum values for the production of the TATP-MIP on the surface of the glassy carbon electrode were: a template concentration of 100 mmol·L^−1^, a functional monomer concentration of 0.1 mol·L^−1^, and 10 scan cycles. The experimental data fit the model with a value for r^2^ = 95%.

Pyrrole is usually the preferred functional monomer for electrochemical polymerisation due to its good solubility in a wide range of solvents. In addition, pyrrole can be electropolymerised from aqueous and non-aqueous solvents making it a versatile functional monomer for molecular imprinting by electropolymerisation [16]. The electropolymerisation of pyrrole varies with the type of solvent, type of oxidising reagent used, type of electrode, electrochemical cell type, temperature, pH, and applied electrode potential. The oxidation potential of pyrrole is lower than the oxidation potentials of most common solvents hence electropolymerisation of pyrrole can be easily initiated by application of a suitable potential, usually slightly greater than +0.60 V. During the electropolymerisation process the polymer film grows on the glassy carbon surface blocking the electron exchange with TATP molecules and trapping them in the polymer matrix.

**Figure 5.**
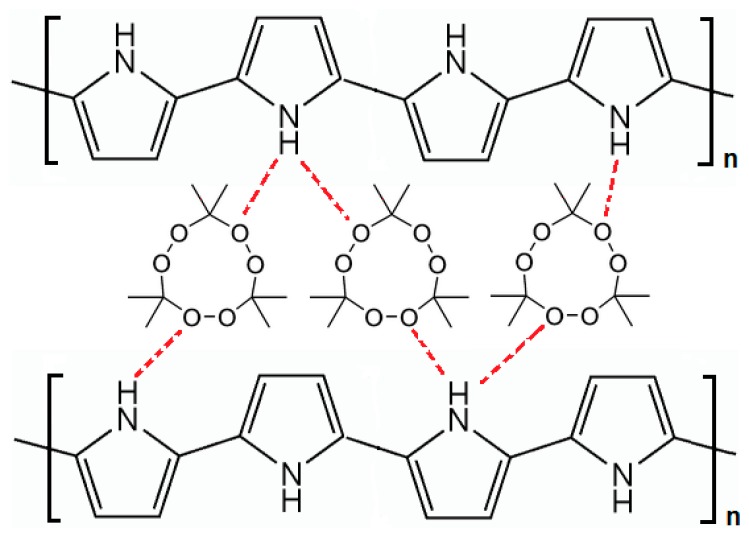
Schematic representation to describe the hydrogen bonding interactions between the TATP molecule and pyrrole units during template trapping in molecular imprinting.

The TATP template molecules are trapped in the polymer matrix as a result of the intermolecular interaction of these molecules with the pyrrole units of the polymer. The technique used follows one of the most typical strategies in developing artificial receptors to produce non-covalent imprinting. Electrostatic interaction and hydrogen bonding are responsible for the possible interaction of the template molecules with the pyrrole unit of the polymer, which leads to the trapping of the template [17]. As shown in Figure 5, the oxygen atoms in the peroxide linkage C-O-O-C of the TATP molecule could form hydrogen bonds with the hydrogen atom in the N-H group of the pyrrole units of the polymer. Chain-branching and cross-linking in the polypyrrole polymer structure generate a three-dimensional matrix with cavities containing the template TATP. This molecular imprinting process creates a complementary microenvironment for the recognition of TATP molecules which is based on the shape, stereochemistry, molecular size, and positioning of the functional groups.

### 3.2. Method Validation

The optimised TATP-Molecular Imprinted Polymer sensor (TATP-MIP) was built as described under materials and methods and its analytical characteristics tested and optimised. In order to better appreciate the advantages of using a modified electrode we compared it with the results obtained for a bare glassy carbon. Table 1 shows the main analytical characteristics for the TATP-MIP sensor and the bare glassy carbon electrode. The TATP-MIP sensor showed ppb detection and quantification limits, which makes the modified electrode suitable for trace analysis. When compared to those obtained for glassy carbon (GC), the values obtained were 4200 μg·L^−1^ and 12,300 μg·L^−1^ for the limit of detection and quantification, respectively. Those values are well within ppm levels, which make the GC electrode less suitable for trace level analysis.

The linearity range is also wide enough for the detection of TATP at trace levels and the correlation coefficient obtained for the calibration curve was also good. The range obtained for the GC electrode was 12,300–222,500 μg·L^−1^, again well within mid ppm levels, and a correlation coefficient r^2^ = 0.966.

The values obtained for precision are extremely good. The precision, expressed as %RSD, was slightly above 1% in the less favorable case (1108 μg·L^−1^). For a GC electrode, the %RSDs obtained for TATP solutions of 88,850 and 177,700 μg·L^−1^ were 3.74% and 2.33%, respectively.

**Figure 6.**
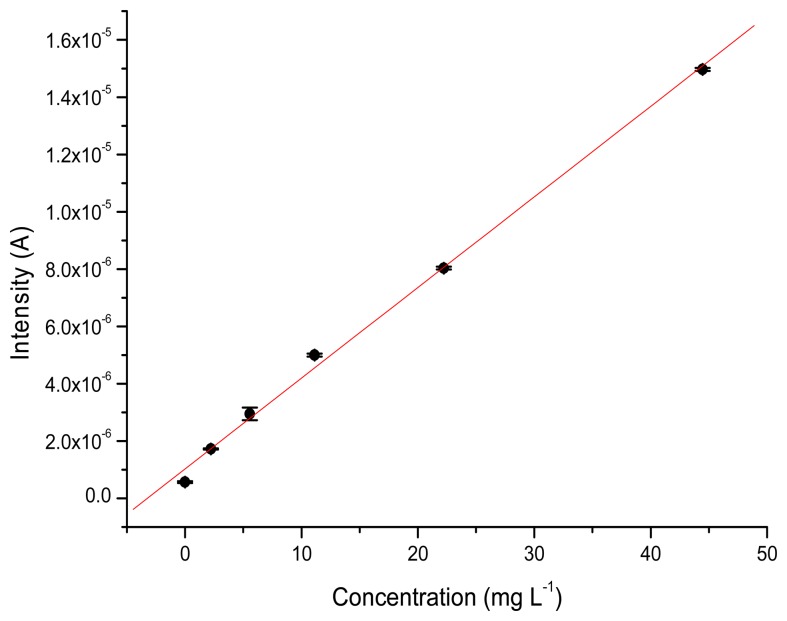
Calibration curve obtained for the MIP-TATP electrode.

**Figure 7.**
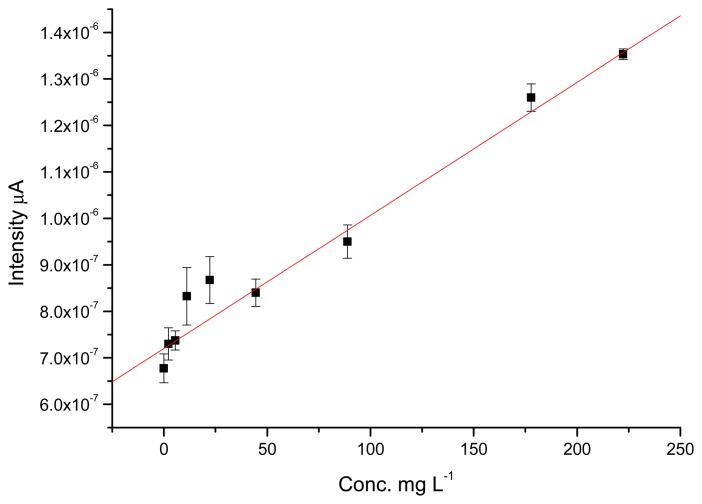
Calibration curve obtained for the bare glassy carbon-TATP electrode.

The values for recovery are also good, with values ranging from 90% to 100%. For the GC electrode, the percentage of recovery obtained for 88,850 and 177,700 μg·L^−1^ TATP solutions were 98.9% and 93.7%, respectively. In this case the GC electrode performed slightly better than the TATP-MIP. Figures 6 and 7 show the calibration curves obtained for the MIP-TATP and the TATP-bares glassy carbon sensor, respectively.

The last part of the validation protocol involved a study of interferences to evaluate the selectivity of the different electrodes in the presence of other substances likely to be found in an explosive mixture. For example, the Al-Qaeda terrorist Richard Reid, commonly known as ‘the shoe bomber’, attempted to ignite an explosive powder mixture of TATP and PETN packed in his shoes on American Airlines Flight 63 on December 2001 [3,18]. IEDs made of a mixture of explosives containing TATP require of a special analytical procedure compared to mixtures without TATP. In this case, PETN is an explosive that can be detected using screening methods for nitro explosives whereas TATP contains no nitro groups and would remain undetected for this procedure.

**Figure 8.**
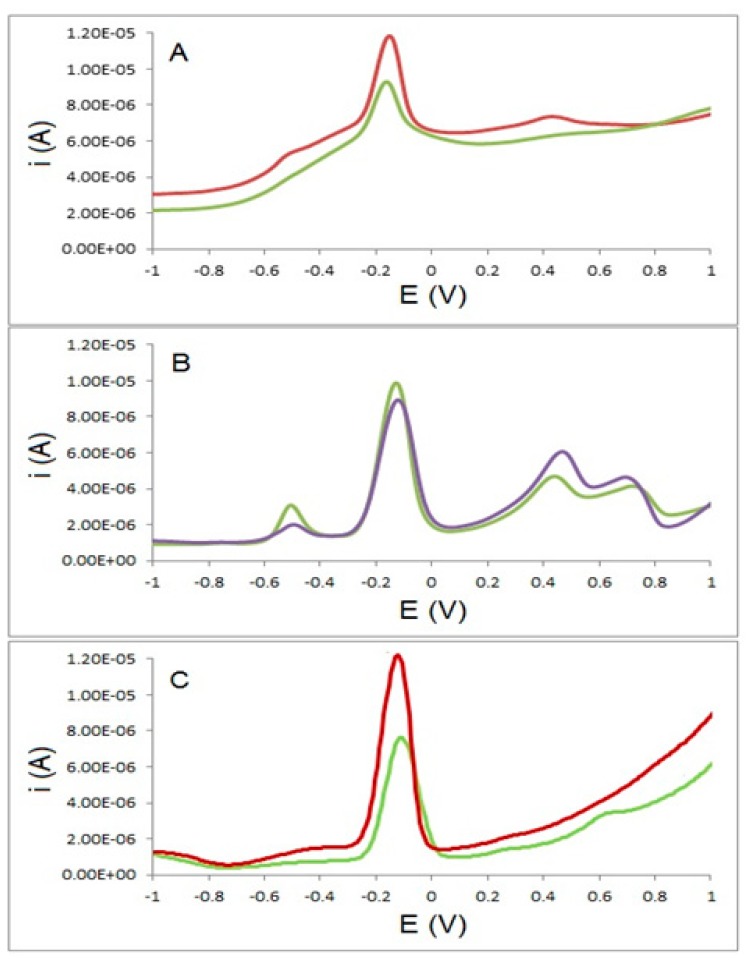
Differential pulse voltammograms of (**A**) TATP at concentrations of 1108 and 2216 μg·L^−1^ (green and red colors, respectively) and using a molecular imprinted polymer modified electrode and (**B**) TATP at concentrations of 44.42 and 88.85 mg·L^−1^ (blue and green colors, respectively) using a bare glassy carbon electrode. For (A) and (B) the solutions contained 1 mg·L^−1^ of each of PETN, TNT, RDX, and HMX with 0.025 mol·L^−1^ LiClO_4_ as supporting electrolyte. For (**C**) the solution contained TATP at concentrations of 2216 and 4432 μg·L^−1^ (green and red colors, respectively) and 0.025 mol·L^−1^ LiClO_4_ using a molecular imprinted polymer.

In order to demonstrate the selectivity of the sensor and to explore potential interferences from other explosives, solutions containing 1 mg·L^−1^ of each of PETN, TNT, RDX, and HMX, in 0.025 mol·L^−1^ LiClO_4_ as the supporting electrolyte and different concentrations of TATP were prepared and analysed using both electrodes. Figure 8 shows the results of these analyses in the range of potentials where TATP is electroactive. It is clear that, in the case of the TATP-MIP electrode, only a single peak corresponding to TATP is observed. In the case of the GC electrode multiple peaks are observed from the reduction and oxidation of the different explosives. In both cases the identification of TATP is clear as both peaks around −0.15 V are clearly defined.

For the TATP-MIP electrode, percentage recovery values of 108% and 77% were obtained for TATP concentrations of 11.1 and 22.2 μg·mL^−1^ respectively. For the GC electrode, percentage recovery values in excess of 540% and 400% were obtained for TATP concentrations of 88.85 and 177.7 μg·mL^−1^, respectively. In this case it is clear that the presence of these interfering explosives in similar concentrations to those of TATP dramatically affected the performance of the GC electrode but did not significantly affect the results obtained for the TATP-MIP electrode.

It has been demonstrated that the molecularly imprinted polymer both enhances and improves the electrochemical response when compared to a glassy carbon electrode for the analysis of TATP. There is an increase in the selectivity, sensitivity, precision and accuracy of the determinations for the former. The sensor developed here has proven superior sensitivity compared to those methods based on the use of IMS [6], piezoelectric sensors [9] and antibody-based sensors [8]. It presents slightly lower sensitivity than chromatographic methods coupled to mass spectrometry [1,7], but presents the advantages of lower analysis times, cheaper price and greater portability. When compared to other electrochemical sensors, it presents much lower sensitivity than sensors based on the indirect determination of TATP using H_2_O_2_ [11]. However, these are prone to a high rate of false positives as the determination is not selective enough. When compared to other more selective chronoamperometric methods [13] the sensitivity obtained is similar.

## 4. Conclusions

Unlike other previously developed electrochemical sensors which rely on the indirect determination of TATP, the electrochemical sensor based on a TATP molecular imprinted polymer developed in this study shows high selectivity in the direct determination of TATP in acetonitrile and detection and quantification limits in the range of low parts per billion. The linear response obtained makes this sensor suitable for trace analysis. The method developed using the TATP-MIP sensor has also proven to be both precise and accurate and the selectivity achieved in the presence of other interferences/explosives is superior to that of the glassy carbon electrode alone.

## Figures and Tables

**Table 1.** Method validation results for the determination of TATP using a MIP electrode and glassy carbon (GC) (given in different units).

		**TATP-MIP**	**TATP-Glassy Carbon**
Correlation coefficient (*r^2^*)		0.9964 (*n* = 6)	0.9663 (*n* = 9)
Linear range		82–44,300 μg·L^−1^	12,300–222,500 μg·L^−1^
Reduction potential *E* (*V*) (*vs.* Ag/AgCl)		−0.15	−0.19
Limit of detection *		27 μg·L^−1^	4,200 μg·L^−1^
Limit of quantification *		82 μg·L^−1^	12,300 μg·L^−1^

Recovery (%)	1,108 μg·L^−1^	90.2%	98.9 (88,850 μg·L^−1^) **

	2,216 μg·L^−1^	100.1%	93.7 (177,700 μg·L^−1^) **

Repeatability (%RSD)	1,108 μg·L^−1^	1.098 (*n* = 6)	3.74 (88,850 μg·L^−1^) **

	2,216 μg·L^−1^	0.55 (*n* = 6)	2.33 (177,700 μg·L^−1^) **

* Limits of detection and quantification are calculated as LoD = 3.3SD and LoQ = 10SD blank;

** Concentration of the determination in brackets for GC.
